# Calcium channels as therapeutic targets in head and neck squamous cell carcinoma: current evidence and clinical trials

**DOI:** 10.3389/fonc.2024.1516357

**Published:** 2024-12-20

**Authors:** Jinye Lin, Xijia Wang, Shibo Ma, Dunhui Yang, Kang Li, Dongcai Li, Xianhai Zeng

**Affiliations:** Department of Otolaryngology, Longgang Otolaryngology hospital & Shenzhen Key Laboratory of Otolaryngology, Shenzhen Institute of Otolaryngology, Shenzhen, China

**Keywords:** HNSCC, calcium channels, therapy, calcium signaling, TRP

## Abstract

Head and neck squamous cell carcinoma (HNSCC) originates from the mucosal epithelium of the oral cavity, pharynx, and larynx, and is marked by high rates of recurrence and metastasis. Calcium signaling is associated with the progression of HNSCC and the development of drug resistance. Changes in calcium ion flow can trigger severe pathophysiological processes, including malignant transformation, tumor proliferation, epithelial-mesenchymal transition, and apoptosis evasion. Calcium channels regulate and facilitate these processes. Remodeling of calcium signaling has become one of the most prevalent adaptive mechanisms in cancer cells. Preclinical and clinical evidence indicates that alterations in calcium signaling are crucial for the progression of HNSCC. This review examines the role of calcium channels in HNSCC development and evaluates current clinical trials targeting these channels to assess the feasibility of calcium signaling-based therapies for HNSCC.

## 1 Introduction

Ca^2+^ acts as a second messenger in various cellular processes (1, 2). Increasing evidence suggests that Ca^2+^ signaling is involved in various diseases, including cancer, autoimmune diseases, and viral infections. Mutations, abnormal expression, regulation, or subcellular targeting of Ca^2+^ handling/transport proteins in cancer can distort Ca^2+^ signaling, dysregulating Ca^2+^-dependent effectors and promoting cancer pathophysiology. Cell proliferation, angiogenesis, invasion, and metastasis are important for tumor development (3). Abnormal Ca^2+^ signaling contributes to malignant phenotype development. To achieve rapid proliferation, increased cell motility and invasion, evasion of cell death, evasion of immune attack, or the formation of new blood vessels, tumors remodel their Ca^2+^ signaling networks. Tumorigenic pathways are increasingly linked to changes in the expression or activation of Ca^2+^ channels, transport proteins, or Ca^2+^-ATPases (4, 5).

Head and neck cancer continues to be a leading cause of cancer-related mortality among newly diagnosed cases globally each year. Despite advancements in treatment, around 40% of newly diagnosed patients succumb to this disease (6). Head and neck squamous cell carcinoma (HNSCC), the most common malignant tumors in the head and neck region, originate from the mucosal epithelium of the oral cavity, nasopharynx, oropharynx, hypopharynx, and larynx. Oropharyngeal and laryngeal cancers are typically linked to smoking and heavy alcohol use, whereas pharyngeal cancer is increasingly associated with human papillomavirus (HPV) infection (7). HNSCC is a type of adult cancer. The median age at diagnosis is 66 years for HPV-negative HNSCC, 53 years for HPV-positive HNSCC, and 50 years for EBV-positive HNSCC (8). Regardless of environmental or viral causes, men have a significantly higher risk of developing various forms of HNSCC than women. The typical symptoms of HNSCC vary based on the primary tumor’s anatomical location and its cause, such as environmental carcinogens, HPV, or EBV (9). Advances in biotechnology, surgery, and radiation therapy are improving the prognosis of HNSCC patients, particularly with the inclusion of immune checkpoint inhibitors. Ongoing clinical trials and advancements in precision medicine are progressing rapidly (7).

In the development of treatments for HNSCC we have gained a better understanding of changes in the genome, proteome, microbiome, and metabolome, which helps us move closer to personalized therapy. Our current understanding of the disease not only considers endogenous changes as key factors in tumorigenesis but also takes into account the microenvironmental factors that contribute to carcinogenic mechanisms (10). There is still a limited understanding of the mechanisms of HNSCC development. Calcium channels were first reported in prostate cancer, where it was found that blocking of calcium channel could affect the progression of cancer (11). Therefore, reshaping these dysregulated Ca^2+^ characteristics could be a potential target for cancer treatment (12). Recent advances in calcium channel research have provided new directions for more accurately diagnosing and treating HNSCC. This review delineates the mechanisms of calcium channels in HNSCC development, highlighting recent findings on drugs and methods targeting specific calcium channels for HNSCC treatment.

## 2 Altered cellular calcium transport systems in HNSCC

**Figure 1:**
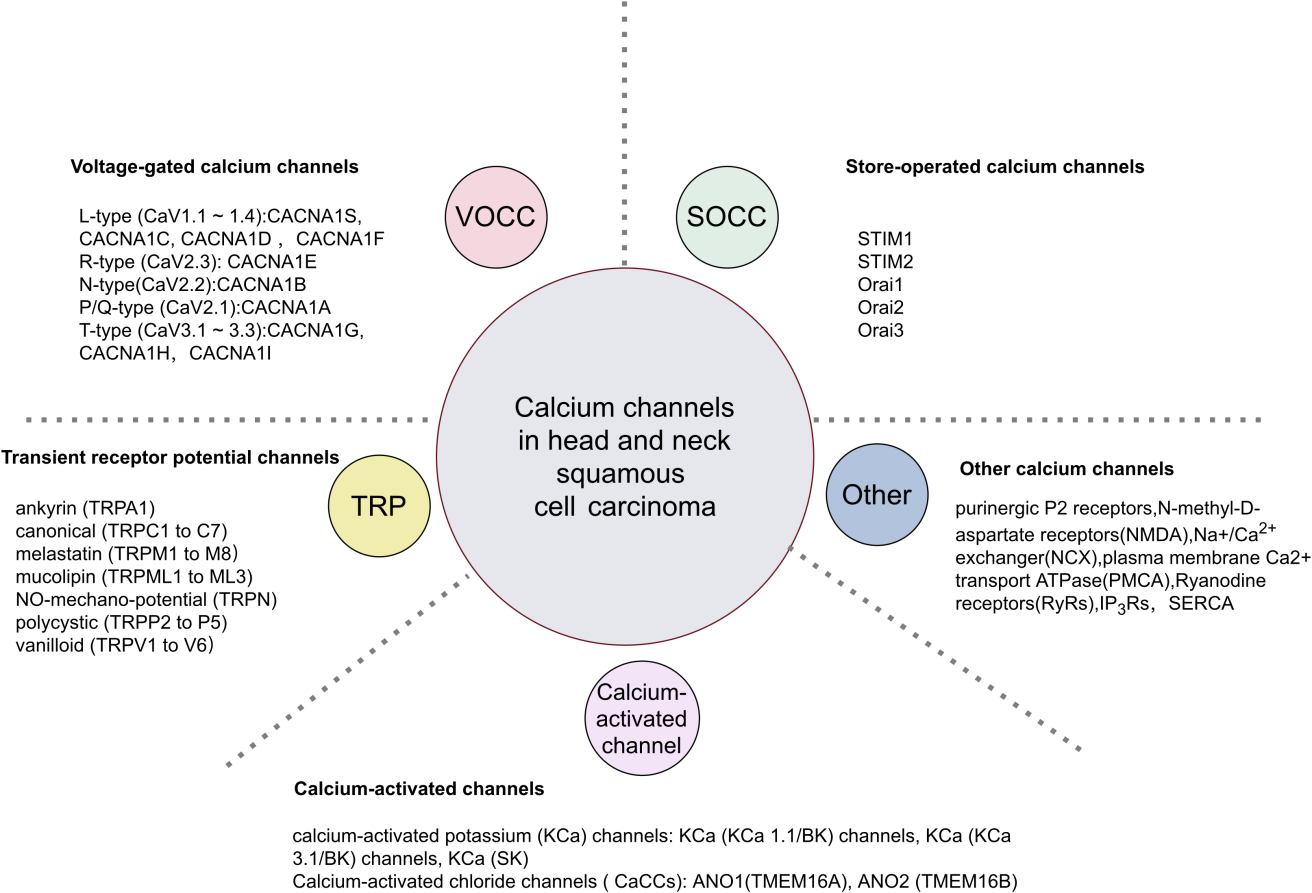
Classification of calcium channels in HNSCC.

The precise regulation of the calcium transport system governs Ca^2+^ movement between intracellular and extracellular spaces, as well as between the cytoplasm and organelles (**Figure 1**). The plasma membrane calcium transport system comprises calcium release-activated calcium (CRAC) channels, transient receptor potential (TRP) channels and voltage-gated calcium channels (VGCC/Cavs) (13). These channels facilitate the entry of extracellular Ca^2+^ into the cell, leading to elevated intracellular Ca^2+^ levels. CRAC channels, representative of store-operated calcium entry (SOCE) channels, are composed of the ER calcium sensor protein STIM and the plasma membrane Orai ion channels (**Table 1**). Calcium channels like inositol 1,4,5-trisphosphate receptors (IP_3_Rs) and ryanodine receptors (RyRs) in the endoplasmic reticulum release ER Ca^2+^ into the cytoplasm, raising intracellular Ca^2+^ levels. The elevated intracellular Ca^2+^ can further activate calcium release channels. The plasma membrane calcium pump (PMCA) and the sarcoplasmic/endoplasmic reticulum calcium pump (SERCA) regulate Ca^2+^ levels by expelling it from the cell or sequestering it into the ER. The sodium-calcium exchanger (NCX) expels Ca^2+^ from the cell, while the mitochondrial Ca^2+^ uniporter (MCU) absorbs Ca^2+^ into the mitochondria, thus maintaining low cytoplasmic calcium levels. We will focus on discussing the regulation of several important channels, transporters, or Ca^2+^ ATPases in HNSCC.

**Table 1:** Altered Calcium Channels in HNSCC.

Channels	Expression level	Tissue type	ref.
Cav3.1	↑	OSCC	(14)
CACNA2D1	↑	LSCC	(15)
TRPM7	↑	NPC	(16)
TRPM7	↑	LSCC	(17)
TRPM6	↑	OSCC	(18)
TRPV1	↑	TSCC	(19)
TRPV1-4	↑	OSCC	(20)
TRPV4	↑	NPC	(21)
TRPV4	↑	OSCC	(22)
TRPA1 and TRPV1	↑	OSCC	(23)
TRPA1	↑	NPC	(24)
TRPC1	↑	TSCC	(25)
TRPC1	↑	TSCC	(26)
TRPP2	↑	LSCC	(27)
TRPP2	↑	NPC	(28)
Orai-1 and Orai-2	↑	OC	(29)
Orai3	↑	OSCC	(30)
Orai1 and STIM1	↑	OSCC	(31)
STIM1	↑	HNSCC	(32)
STIM1	↑	NPC	(33)

↑ means up-regulation.

### 2.1 VGCCs in HNSCC

Recent evidence increasingly highlights the role of VGCC in tumor genesis and progression. VGCCs are classified as low voltage-activated (LVA) or high voltage-activated (HVA) based on their activation threshold. The isomer codes are as follows: L-type (CaV1.1~1.4) encoded by CACNA1S, CACNA1C, CACNA1D, and CACNA1F; R-type (CaV2.3) by CACNA1E; N-type (CaV2.2) by CACNA1B; P/Q-type (CaV2.1) by CACNA1A; and T-type (CaV3.1~3.3) by CACNA1G, CACNA1H, and CACNA1I (13).

Previous research suggests that Cav3.1 significantly influences the proliferation and anti-apoptotic activity of human oral squamous cell carcinoma (OSCC) (34). Cav3.1 expression was overexpressed in OSCC tissues and significantly correlated with Ki-67, PCNA, and Bcl-2 levels (14). In a study, Cav1.2 was found to be strongly enriched in ameloblastoma (AM) by comparative transcriptome analysis. Cav1.2 primarily mediates Ca^2+^ influx in ameloblastoma cells, as demonstrated by the use of agonists and blockers. Genetic study of Cav1.2 demonstrated its direct role in cell proliferation by regulating the nuclear translocation of nuclear factor of activated T cells (NFAT) 1. Cav1.2 is a potential therapeutic target for inhibiting invasiveness during AM progression, necessitating a deeper understanding of the regulatory mechanisms mediating Ca^2+^ signaling invasiveness through Cav1.2 (35).

α2δ1 is a voltage-gated calcium channel subunit, an essential auxiliary unit of CaV1 and CaV2.In previous studies, it has been demonstrated that α2δ1 plays a crucial role in the regulation of CSC calcium oscillations (36), and α2δ1 has been reported in both hepatocellular carcinoma and non-small cell lung cancer cells. α2δ1 subunit is a functional marker and a therapeutic target for non-small cell lung cancer and hepatic tumor-initiating cells (37). A study suggests that α2δ1 has a significant effect on sphere formation or tumorigenicity of laryngeal squamous cell carcinoma (LSCC) both *in vivo* and *in vitro* and has the potential to be a tumor stem cells marker for LSCC, and CACNA2D1 is the coding gene of α2δ1 (38). It was found that overexpression of miR-107 not only reduced the expression level of CACNA2D1 gene, but also inhibited the level of CACNA2D1 protein α2δ1, which could significantly inhibit the malignant biological characteristics of LSCC cells. Thus miR-107 and CACNA2D1 may be potential targets for LSCC prognosis and treatment (15). Therefore, more studies are needed to fully understand the role of VGCCs in HNSCC.

### 2.2 TRP family channels in HNSCC

TRP channels, initially identified as a visual mutant in Drosophila, have been demonstrated since the 1990s to exist in various cell line types and healthy human tissues (39, 40). Seven subfamilies based on their gene sequence names as follows: ankyrin (TRPA1), canonical (TRPC1 to C7), melastatin (TRPM1 to M8), mucolipin (TRPML1 to ML3), NO-mechano-potential, NOMP (TRPN), polycystic (TRPP2 to P5) and vanilloid (TRPV1 to V6) (41). TRP channels, primarily situated on the cell surface, engage with various physiological signaling pathways. Gene expression studies indicate that TRP channels play a role in the pathogenesis of HNSCC, with significant implications for diagnosis, prognosis, and treatment (6).

The TRPM subfamily, comprising TRPM1 to TRPM8, is the largest within the TRP superfamily. Multiple studies indicate that TRPM7 is involved in tumorigenesis and various tumor characteristics, including proliferation, migration, invasion, and metastasis. László Köles et al. reported that TRPM7 is expressed in nasopharyngeal, laryngeal, and hypopharyngeal carcinomas (42). Overexpression of TRPM7 can significantly enhance the migratory ability of non-metastatic nasopharyngeal carcinoma cells. The TRPM7 channel and TRPM7-mediated Ca^2+^ influx may be crucial for the migration of nasopharyngeal carcinoma cells (43). Studies suggest that nasopharyngeal carcinoma patients with TRPM7 overexpression have significantly shorter survival times compared to patients with low TRPM7 expression. TRPM7 is involved in the metastasis of nasopharyngeal carcinoma by enhancing the invasion and migration of nasopharyngeal carcinoma cells (16). TRPM7 regulates tumor proliferation by continuously activating the JAK2/STAT3 signaling pathway. Compared to high TRPM7 expression, nasopharyngeal carcinoma patients with low TRPM7 expression have higher survival rates. Knocking out the TRPM7 gene can increase the sensitivity of nasopharyngeal carcinoma patients to radiotherapy (44). TRPM7 is partially involved in the growth and proliferation of human head and neck tumor cell lines (45). In patients with laryngeal squamous cell carcinoma, circRNAs were first discovered to regulate the expression of TRPM7 (17). Silencing the expression of TRPM7 can significantly inhibit the metastasis of HNSCC cells, reducing their migration and invasion abilities. TRPM7 is crucial for maintaining HNSCC stem cell characteristics and contributes to chemotherapy resistance. Silencing TRPM7 can inhibit multiple oncogenic signaling pathways and reduce the migration, invasion, colony formation, and tumor sphere formation of human squamous cell carcinoma cells in culture (46). Research has shown that the anesthetic drug midazolam can inhibit the growth and proliferation of human hypopharyngeal squamous cell carcinoma cells by suppressing the expression of TRPM7 (47).

TRPM2 and TRPM6 are expressed in human oral squamous cell carcinoma (18). Research indicates that the functions of TRPM2 differ between the membrane and the nucleus. Elevated membrane TRPM2 levels protect against early tumor growth. In the later stages, the loss of membrane TRPM2 and the increase of nuclear TRPM2 enhance the susceptibility to tumorigenesis (48). Menthol enhances the invasion of oral squamous cell carcinoma cells, while TRPM8 antagonists inhibit this invasion by blocking both menthol-induced and inherent TRPM8 activity (49).

Some studies suggest that TRPV1 is expressed in human OSCC and precancerous lesions. The increased expression of TRPV1 is not related to the malignancy level of the tumor but represents an early step of molecular overexpression in the tumorigenesis process (19). TRPV1-4 expression was confirmed with higher levels observed in patients with OSCC compared to normal epithelium. Alcohol consumption and smoking are linked to oral cancer and have been shown to elevate TRPV1-4 receptor expression in normal oral mucosa (20). Capsaicin, a natural TRPV1 agonist, and Capsazepine, a synthetic TRPV1 agonist, are reported to exhibit no cytotoxic effects on non-malignant cells *in vitro* (50). Elevated TRPV2 expression correlates with poor prognosis in HNSCC patients, while ANXA6 facilitates autophagy and lymphatic metastasis in HNSCC by modulating TRPV2 expression through mTOR phosphorylation inhibition (51).

Research has found that TRPV4 expression is significantly elevated in nasopharyngeal carcinoma tissues and human nasopharyngeal carcinoma cell lines.TRPV4 stimulates tumor occurrence through Ca^2+^/NFAT4-signaling (21). Higher expression of TRPV4 is also found in OSCC. TRPV4 induces protein kinase activity that regulates cancer cell growth via activation of AKT (22). TRPV4 is present in tongue squamous cell carcinoma cells and primary afferent fibers, with cancer pain intensity correlating to the extent of TRPV4 phosphorylation by protease-activated receptor 2 (52).

TRPA1 expression is markedly enhanced in nasopharyngeal carcinoma and OSCC (23). Notably, OSCC cells can release lipids that activate TRPV1 (53) and TRPA1 receptors on sensory neurons, leading to pain associated with oral cancer. The up-regulation of TRPA1 protein is associated with the progression of nasopharyngeal carcinoma (24).

Compared with adjacent tissues, the expression of TRPC1 is increased in tongue squamous cell carcinoma tissues, thus having a clinical role in distinguishing and predicting tumor risk (25). The expression of TRPC1 is positively correlated with the tumor-node-metastasis staging and the depth of invasion in tumor patients. Research indicates that TRPC1 knockdown inactivates the PI3K/AKT pathway, thereby reducing the proliferation and invasion of tongue squamous cell carcinoma cells (26). The migratory capacity of nasopharyngeal carcinoma cells is associated with TRPC1 expression, and TRPC1 silencing can decrease cancer cell migration (54). Knock down the expression of TRPC6 located at 11q22 can significantly inhibit the invasion of HNSCC cells. Additionally, amplification and overexpression of TRPC6 have been observed in HNSCC tumor samples (55).

Encoded by the PKD2 gene, TRPP2 is a non-selective cation channel that control calcium signaling (56). Elevated TRPP2 expression may expedite metastasis in human laryngeal squamous cell carcinoma cells via epithelial-mesenchymal transition (EMT) (27). Further research indicates that TRPP2 knockout enhances cell proliferation by downregulating the PERK/eIF2α pathway, while the AMPK/ACC pathway activates cell proliferation through feedback mechanisms (57). The research suggests that cancer cells can effectively absorb exosome/TRPP2 complexes, significantly inhibiting the expression, migration, and invasion of TRPP2 in cancer cells (58). TRPP2 is also highly expressed in nasopharyngeal carcinoma, promoting its progression by upregulating the Skp2/c-Myc pathway (28). However, due to the wide tissue distribution and multiple functions of TRP channels, identifying specific TRP channels and their selective cell localization associated with specific cancer-promoting functions is critical for the development of safer and better anticancer drugs.

### 2.3 Store-operated calcium channels in HNSCC

Store-operated channels are plasma membrane Ca^2+^ ion channels regulated by the Ca^2+^ content within the endoplasmic reticulum (ER). They are primarily identified as components of a biphasic Ca^2+^ signaling mechanism, which includes intracellular Ca^2+^ release and Ca^2+^ entry via plasma membrane channels (59). In 1983, it was theoretically proven that receptor-activated Ca^2+^ release primarily involves the second messenger inositol 1,4,5-trisphosphate (IP_3_). A decrease in ER Ca^2+^ concentration activates STIM1 and STIM2. The conformational changes in STIM1 and STIM2 stimulate Orai and TRPC channels (60). The main proteins functioning in CRAC channels have been identified as the ER Ca^2+^ sensors STIM and the CRAC channel subunits Orai, which are crucial for the proliferation, migration, metastasis, and apoptosis of cancer cells (61).

The Orai channel family, comprising Orai1, Orai2, and Orai3, is characterized by significant calcium selectivity. Orai1, the most extensively studied among the three Orai homologs, plays a critical role in cancer progression (62). Orai1 is a newly identified molecular regulator of carcinogenicity and stemness in OSCC. Orai1 promotes OSCC stemness through the activation of the Ca^2+^-dependent transcription factor NFAT. The Orai1/NFAT pathway regulates hematopoietic stem cells.Orai1 regulates Ca^2+^ signaling in OSCC cells and mitigates nociceptive pain and hyperalgesia by controlling collagenase expression among matrix metalloproteinases when deficient (63, 64). Additionally, Orai2 is overexpressed in OSCC tissues, significantly contributing to cell proliferation, survival, migration, and metastasis (29). Orai3 promotes cancer stem-like cell phenotypes by upregulating the stemness transcription factor ID1, suggesting that the Orai3/ID1 axis is a novel regulatory mechanism for maintaining cancer stemness in OSCC (30). Studies have shown that activation of prostaglandin receptor 4 (EP4) promotes cell migration via PI3K, and the migration of oral cancer cells is regulated by the EP4/PI3K/Orai1 signaling pathway (65). Studies have shown that not only Orai1 but also STIM1 expression is significantly increased in OSCC (31).

Studies have shown that HNSCC exhibits overexpression of STIM1 in tumor tissues and is involved in the growth and anti-apoptotic processes of HNSCC, but it is not related to neck lymph node metastasis (32). Silencing STIM1 inhibits hypopharyngeal carcinoma cell growth and induces cell cycle arrest and apoptosis (66). Nasopharyngeal carcinoma (NPC), a head and neck malignancy, is linked to the Epstein–Barr virus (EBV) (67). EBV manipulates the EMT of NPC cells to promote metastatic potential by enhancing STIM1 signaling. Inhibition of STIM1 signaling can suppress the *in vivo* spread and lymphatic metastasis of NPC cells (33). Further investigation into the upstream signaling pathways of STIM1 expression indicates that the miRNA-185-5p/STIM1 axis influences the invasiveness of NPC cell lines by modulating cell adhesion through epidermal growth factor receptor (EGFR) activation (68). EBV enhances EGF-induced STIM1/ERK1/2 signaling. Blocking this signaling pathway may inhibit EBV-enhanced angiogenesis in NPC (69). Blocking exosome-mediated EBV-associated oncogenic signaling molecules could be an effective strategy for treating NPC (70). Increasing a long non-coding RNA can inhibit autophagy in cancer cells, and upregulating the PTBP1/STIM1 axis promotes the stemness of nasopharyngeal carcinoma cells (71). Taken together, the introduction of drugs that specifically target SOCE would be a viable and practical strategy for HNSCC therapy.

### 2.4 Other calcium mediators in HNSCC

N-methyl-D-aspartate receptors (NMDA) purinergic P2 receptors also regulate Ca^2+^ influx. PMCA and NCX mediate the extrusion of Ca^2+^. SERCA pump, IP_3_Rs and RyRs control the movement of Ca^2+^ within the ER (11).

Research indicates that P2R signaling directly affects pro-inflammatory cytokine production in human OSCC cell lines and promotes cancer progression in healthy host tissues (72). NMDAR1 is overexpressed in OSCC and is significantly associated with tumor size, lymph node metastasis, and cancer staging (73). Research demonstrates that salinomycin effectively eliminates cancer stem cells *in vivo* and *in vitro*, including in HNSCC cells (74). Salinomycin’s neurotoxicity is driven by elevated Na^+^ levels, which subsequently increase membrane Ca^2+^ via Na^+^/Ca^2+^ exchangers in both the plasma membrane and mitochondria (75). Inhibiting the mitochondrial Na^+^/Ca^2+^ exchanger can partially mitigate salinomycin-induced neurotoxicity *in vivo* without compromising its antitumor efficacy (76). Ca^2+^-ATPases, part of the P-type ATPase superfamily, are categorized into three subtypes based on subcellular localization: plasma membrane Ca^2+^-ATPase (PMCA), ER/SR Ca^2+^-ATPase (SERCA), and Golgi/Golgi-derived vesicles secretory pathway Ca^2+^-ATPase (SPCA) (77). PMCA1 is abundantly expressed in normal oral epithelial cells but reduced or absent in OSCC cell lines (78). The reduced expression of ryanodine receptor 2 (RYR2) in tissues adjacent to tumors and in precancerous lesions may be a risk factor for poor prognosis and impending malignant transformation (79). C17orf104, ITPR3, and DDR2 are among the genes frequently mutated in multiple metastatic or recurrent head and neck squamous cell carcinomas (80).

### 2.5 Calcium-activated channels in HNSCC

Calcium-activated potassium (KCa) channels are categorized by their single-channel conductance into large conductance (KCa1.1/BK), intermediate conductance (KCa3.1/IK), and small conductance (SK) channels (81, 82). Restoring KCa3.1 activity in HNSCC CD8^+^ T cells can counteract the inhibitory effects of adenosine. Compared to A2AR receptor inhibition, KCa3.1 activation therapy has greater advantages because it can simultaneously counteract multiple immunosuppressive factors within the tumor microenvironment (TME). Activating KCa3.1 channels may enhance immune surveillance and improve cancer immunotherapy response (83).

Calcium-activated chloride channels (CaCCs) include ANO1 (TMEM16A) and ANO2 (TMEM16B) from the Anoctamin family (84). The amplification and overexpression of ANO1 and other genes on 11q13 are linked to a higher incidence of future metastases in HPV-negative HNSCC. Functional synergy among these genes may explain their frequent co-amplification at the 11q13 locus (85). ANO1 is not only a new tumor therapeutic target, but also a predictor of drug therapeutic efficacy (86). Further studies indicate that TMEM16A is more crucial in HPV-negative HNSCC compared to HPV-positive HNSCC (87). In HNSCC, the expression of ANO1 is epigenetically regulated through promoter methylation. ANO1 is considered a major driver of the “growth” or “death” pattern in the progression of HNSCC. ANO1 gene amplification frequently occurs in premalignant lesions and invasive tumors (88).

## 3 Drugs targeting Ca^2+^ channels/transporters/pumps for cancer treatment

Mainstream anticancer chemotherapy drugs primarily target DNA replication, DNA/RNA synthesis, DNA damage, and growth factor receptor signaling. Currently, mainstream cancer chemotherapy drugs do not target the Ca^2+^ signaling mechanism, possibly because the expression of Ca^2+^ channels and transporters on the cell surface can be easily exploited by new drugs or even antibody therapies. Ca^2+^ signaling proteins lack specificity, and therapies targeting them might produce unacceptable adverse effects (89). Recently, novel therapies targeting calcium transporters, channels and pumps are used to treat cancers (11, 90), and the related ongoing clinical trials can be observed (**Table 2**).

**Table 2:** Calcium-signaling targeting drugs under development for cancer therapeutic purposes.

Target	Targeting drugs/therapy method	Mechanisms	Trial phase	Trial	Cancer type	Refs.	Clinical Trials. gov Identifier
TRPV6	SOR-C13	Inhibits the calcium uptake via TRPV6, reducing cell proliferation	I	Advanced tumors of epithelial origin	metastatic epithelial ovarian, pancreatic and prostate cancers	(91)	NCT01578564
TRPV2	Tranilast	Inhibiting CAF induced adverse reactions in the immune environment	II	Tumor growth after irradiation	nasopharyngeal carcinoma	(92)	NCT05626829
T-type	Mibefradil	Inhibitor of VGCCs	I	Solid cancers	High-grade gliomas	(93)	NCT01480050
SOCE	Carboxyamido-triazole (CAI)	Inhibition of transmembrane calcium influx	II	Relapsed Epithelial Ovarian Cancer	Wide range of solid	(94)	NCT00019461
SERCA	Mipsagargin (G-202)	Inhibition of the SERCA pump induces apoptotic cell death	II	Advanced Hepatocellular Carcinoma	glioblastoma multiforme ,prostate cancer ,malignant glioma, clear cell renal and cell carcinoma	(95)	NCT01777594
PMCA	Calcium electroporation(Ca2+-EP)	Induces a large amount of intracellular calcium influx leading to cell necrosis	I	Recurrent head and neck cancer	Cutaneous metastases , Colorectal cancer, Keloid	(96)	NCT03051269

### 3.1 TRP channel inhibitors

The TRPV6 inhibitor SOR-C13 was evaluated in a Phase I clinical trial (NCT01578564) with 23 patients having advanced solid tumors. Among these patients, one had NPC. The study concluded that SOR-C13 is safe, well-tolerated, and exhibits promising anti-cancer activity, especially in two patients with pancreatic ductal adenocarcinoma (91). Tranilast is the most extensively studied inhibitor of TRPV2 (90, 97). Tranilast has the potential to act as a CAF inhibitor. The inhibition of CAF function by Tranilast can suppress the induction of immunosuppressive cell types *in vitro* (98). The text discusses an ongoing study (NCT05626829) on the use of Tranilast as a radiosensitizer in the reirradiation of NPC. This prospective Phase II interventional trial assesses the safety and efficacy of adding Tranilast for patients with recurrent NPC post-radiotherapy. Studies have found that compared to radiation-sensitive NPC, radiation-resistant cancer tissues are increasingly infiltrated by CAFs. Tranilast treatment demonstrated that CAFs enhance the survival of irradiated NPC cells via the NF-κB pathway, contributing to radiation resistance, which Tranilast can disrupt. This treatment limits the CAF-induced survival of NPC cells and reduces their radiation-resistant characteristics (92).

### 3.2 T-type calcium channel blockers

Blocking T-type Ca^2+^ channels can cause cell cycle arrest and reduce cancer cell proliferation. Inhibiting T-type channels alone is unlikely to completely eliminate cancer cells and would need to be combined with standard cytotoxic chemotherapy or radiation therapy (99). Mibefradil selectively inhibits the T-type calcium channel Cav3, which is primarily involved in calcium influx in various solid cancers, including glioblastoma (100). Previously used primarily for hypertension and angina, this study is the first to explore Mibefradil as an anti-cancer drug in humans. The study verified that sequentially administering Mibefradil and temozolomide in recurrent high-grade gliomas is safe, and that administering Mibefradil four times daily optimizes systemic exposure to near-maximal drug concentration (93). This study also has its limitations. In research where overall survival was used as a secondary endpoint, the observed results showed heterogeneity. The study included temozolomide, a drug known to be active against gliomas, making the extent to which Mibefradil enhanced this activity unclear. Additional efficacy trials are required to validate the therapeutic effects of this regimen (93). ClinicalTrials.gov lists studies on Mibefradil, including NCT01550458, which assesses the safety of administering Mibefradil four times daily in healthy volunteers, and NCT01480050, which explores the combination of Mibefradil Dihydrochloride and Temozolomide for treating recurrent glioma.

### 3.3 SOCE targeted therapy

Carboxyamido-triazole (CAI), a calcium influx inhibitor, exhibits anti-angiogenic and anti-invasive properties, stabilizing tumor progression (101). A Phase I clinical trial for recurrent solid tumors established that the maximum tolerated dose is daily CAI administration combined with Paclitaxel injections every three weeks. The study demonstrated that the combination of these two drugs is well-tolerated, active, and potentially effective against several different types of cancer (102). In the Phase I evaluation of CAI, due to disease stabilization, a Phase II trial was conducted on patients with recurrent ovarian cancer. The trial indicated that CAI shows potential in stabilizing recurrent ovarian cancer with moderate toxicity, warranting further research (94).

### 3.4 SERCA inhibitors

SERCA inhibitors, particularly Thapsigargin (Tg), were initially proposed as novel therapeutic agents for cancer treatment and have been extensively studied. Tg induces apoptosis in cancer cells independently of proliferation by inhibiting the SERCA pump (95). To overcome the high toxicity and non-selectivity of Tg, which disrupts intracellular Ca²⁺ homeostasis in both cancer and normal cells, a Tg-based prodrug strategy has been developed (103). A prodrug is an inactive compound that can be cleaved by proteases specific to cancer cells (104). Prostate specific membrane antigen (PSMA) is a carboxypeptidase that is overexpressed in most endothelial cells within prostate cancer and various other tumor types but is not expressed in normal vascular endothelium. G202 is a Tg-based PSMA-activated prodrug targeting PSMA enzyme activity in tumor vasculature, potentially treating various solid tumors (105).

Currently, G202, under the name mipsagargin, has entered clinical trials. Its cytotoxic activity is concealed by a peptide cleaved by PSMA. In a Phase I clinical trial (NCT01056029) for patients with refractory, advanced solid tumors, mipsagargin exhibited a unique mechanism of action, stabilizing the disease and demonstrating antitumor activity. By using a PSMA-targeted monoclonal antibody combined with the potent microtubule inhibitor MMAE (Monomethyl auristatin E), this approach directly integrates the targeting ability of the antibody with the cytotoxic power of the chemotherapy drug. This provides a precise and effective treatment method with relatively fewer side effects, concentrating the drug’s toxicity on the tumor site and thereby reducing damage to normal cells (105). Building on promising early clinical trial results, a Phase II clinical trial (NCT01777594) was conducted in adult patients with hepatocellular carcinoma. The trial established the maximum tolerated dose of mipsagargin as 66.8 mg/m², administered via a 1-hour intravenous infusion on days 1, 2, and 3 of a 28-day cycle (106). Phase II clinical trials encompassed studies on glioblastoma multiforme (NCT02067156), prostate cancer (NCT01734681), malignant glioma (NCT02876003), and clear cell renal cell carcinoma (NCT02607553), among others (95).

### 3.5 Other drugs

Calcium electroporation (Ca^2+^-EP) is a novel cancer treatment that benefits the treatment of localized tumors by inducing a significant influx of calcium into cells, leading to cell necrosis (107). The endoplasmic reticulum, sarcoplasmic reticulum, and mitochondria act as calcium ion reservoirs. Calcium is transported into the ER and SR by the sarcoplasmic/endoplasmic reticulum Ca^2+^-ATPase (SERCA). Elevated intracellular Ca^2+^ levels can be toxic, leading to ATP depletion and cell necrosis (108). Binding ER with Ca^2+^ can increase and accelerate the concentration of cellular ions within cancer cells. Calcium electroporation can induce cell necrosis by increasing calcium influx, as demonstrated *in vitro* on 18 different cell types, showing effects comparable to bleomycin (108). *In vivo* experiments on mice with five different human tumors showed varying sensitivity to the treatment (109). Research indicates that calcium electroporation has anti-vascular effects on both normal and tumor blood vessels *in vitro* and *in vivo*, and significantly induces tumor necrosis (110). Calcium electroporation elicits distinct effects on cell spheroids: it reduces the size of cancer cell spheroids but does not alter the size of normal cell spheroids (111).

A phase I clinical trial (NCT03051269) on calcium electroporation for head and neck tumors included 6 patients, with no observed hypercalcemia, arrhythmia, or serious adverse events post-treatment. One patient achieved complete clinical remission one year post-treatment. Therefore, the future prospects for calcium electroporation are promising, though larger-scale trials are still needed (96). A phase II study compared calcium electroporation (CaEP) and electrochemotherapy (ECT) for treating skin metastases, including 47 patients, 7 of whom were part of the research protocol. After 6 months of follow-up, calcium electroporation and chemotherapy showed no significant difference in objective response. Seven days after treatment, biopsies collected from tumors treated with either calcium electroporation or electrochemotherapy showed a significant reduction in cancer cell numbers and higher levels of cell death. Compared to electrotherapy, calcium electroporation offers better cosmetic results and is easier to manage due to the lack of cytotoxicity of calcium itself (112).

Extensive research is being conducted on electroporation therapy for head and neck cancer. This nanomedicine and medical technology hold significant clinical potential for cancer treatment (108). Furthermore, calcium electroporation, which has accessibility and the ability to modulate systemic immune responses, is becoming increasingly popular (113). Calcium electroporation has been widely used to enhance the treatment of superficial tumors with chemotherapy, and trials for other internal tumors are ongoing (114, 115). Calcium is more accessible, easier to manage, and can act as a cost-effective, non-toxic substitute for bleomycin (110). In a Phase I study for the treatment of keloids, calcium electroporation led to a reduction in thickness by more than 30% in patients with keloids. The treatment was well-tolerated, with no severe adverse reactions or recurrences observed (116). Calcium electroporation demonstrates therapeutic potential, necessitating further clinical studies to validate its efficacy.

## 4 Conclusions

Calcium signaling is crucial in various cells that facilitate the development and metastasis of HNSCC. Investigating the link between calcium signaling and HNSCC can reveal related genes, pathways, and downstream effectors, emphasizing the clinical importance of altered calcium signaling and identifying new therapeutic targets (6). This emerging evidence suggests a complex relationship between calcium signaling and the clinical progression of HNSCC (11). Targeting calcium signaling mechanisms specifically in malignant cells is challenging due to calcium’s widespread role in most cell types and physiological processes. An ideal therapeutic target should be uniquely expressed by cancer cells or exhibit a distinct gain or loss of function to prevent unacceptable adverse effects. Data on calcium signaling mediators’ expression and function in HNSCC patients will be crucial for developing highly effective drugs with minimal side effects (11, 90). Despite this, many drugs targeting calcium signaling have been developed, though many are aimed at solid tumors in general rather than specifically at HNSCC. Several of these drugs have demonstrated good safety profiles in Phase I/II clinical trials. Most of these drugs have been assessed in diverse and limited patient populations with various malignancies, with few studies specifically including HNSCC patients (89, 90). Calcium channels were expressed in a lot of tissues of the human body, thus, using inhibitors/blockers of calcium channels may cause side effects. Nanomaterial may be a potential strategy for inhibitors/blockers of calcium channels administration to reduce the side effects of targeting calcium channels. Because the nanomaterials can deliver drugs to target tumor cells, which could improve the therapeutic effect by avoiding the delivery of drugs to normal cells. The link between calcium signaling and the reprogramming of cellular energy metabolism remains relatively unexplored. Further research is needed on the possible role of Ca^2+^ signaling in glycolytic regulation, glycolytic conversion, and the use of ATP produced by glycolysis to fuel Ca^2+^ pumps in cancer cells. The relationship between Ca^2+^ signaling and the tumor microenvironment remains unclear. Cancer-associated fibroblasts, for example, are in an “activated” state and interact dynamically with cancer cells, which may be regulated by Ca^2+^ signaling. Undoubtedly, calcium signaling mechanisms are an intriguing target for cancer therapy, but the pharmacological opportunities and clinical benefits provided by calcium signaling still need to be further elucidated.
